# How Recovery Attitudes Relate to Views on Coercive Interventions and Interpersonal Characteristics in Psychiatric Nurses: A Quantitative Cross‐Sectional Study

**DOI:** 10.1002/nop2.70936

**Published:** 2026-10-16

**Authors:** Aimi Kinoshita, Hisato Igarashi, Shiori Ishida, Seiji Shimosato

**Affiliations:** ^1^ Shinshu University School of Health Sciences Nagano Japan

**Keywords:** coercion, helping behaviour, mental health recovery, mental health services, psychiatric nursing

## Abstract

**Aims:**

To examine the associations among psychiatric nurses' attitudes toward mental health recovery, attitudes toward coercive interventions, and interpersonal care characteristics, particularly the dimensions of dominance and coldness.

**Design:**

Cross‐sectional study.

**Methods:**

Self‐administered questionnaires were used to collect demographic information and assess recovery‐oriented attitudes, attitudes toward coercive interventions, and interpersonal characteristics. Intergroup differences were analysed using *t*‐tests and analysis of covariance, with Bonferroni corrections applied, whereas structural equation modelling was used to examine the relationships among recovery‐oriented attitudes, attitudes toward coercive interventions, and interpersonal characteristics.

**Data Sources:**

Survey responses were obtained from 604 nurses working in public psychiatric hospitals in Japan between August and October 2022. Recovery‐oriented attitudes, attitudes toward coercive interventions, and interpersonal characteristics were assessed using established instruments.

**Results:**

Higher recovery‐oriented attitudes were associated with lower levels of stigma‐based evaluations of service users and lower levels of interpersonal coldness, whereas the direct association with dominance was limited. Staff nurses reported higher stigma‐based evaluations and greater interpersonal coldness than head and deputy head nurses, whereas nurses working in forensic psychiatric wards demonstrated higher safety paradigm scores and negative evaluations than those in community support services. Overall, nurses' knowledge of and attitudes toward recovery were associated with their evaluations of service users and interpersonal care styles, even when coercive interventions were occasionally required.

**Conclusion:**

Recovery‐oriented attitudes appear to play a potentially important role in promoting ethical, person‐centred psychiatric nursing.

**Implications for the Profession and/or Patient Care:**

Incorporating recovery‐oriented education into routine psychiatric training may help nurses reduce stigmatizing evaluations. This approach may also decrease interpersonal coldness, thereby fostering empathetic care and preserving service user dignity when coercive interventions are occasionally necessary.

**Impact:**

These findings provide preliminary evidence suggesting that recovery‐oriented attitudes may reduce negative caregiving behaviours, thereby advancing ethical practice and improving the quality of psychiatric nursing.

**Reporting Method (EQUATOR Guideline):**

This study adhered to the Strengthening the Reporting of Observational Studies in Epidemiology reporting guidelines.

**Patient or Public Contribution:**

No patient or public contribution was involved in the design or conduct of this study.

## 1 Introduction

Mental health recovery is defined as a deeply personal process of living a meaningful and hopeful life despite the limitations imposed by mental illness (Anthony 1993; Slade et al. 2014). This concept has increasingly become a guiding principle in international mental health services, and its implementation is widely promoted. In contrast, coercive interventions, such as mechanical restraint (the application of mechanical devices, such as belts, to service users) and seclusion (confining a service user alone in a locked room), continue to be used in psychiatric inpatient settings as safety measures to protect service users and others when deemed unavoidable (Doedens et al. 2020; Savage et al. 2024). In Japan, the frequent and prolonged use of seclusion and mechanical restraint remains a major concern (Savage et al. 2024). These interventions have been associated with adverse psychological outcomes, including fear, anxiety, and posttraumatic stress disorder (Aguilera‐Serrano et al. 2018). Moreover, they are considered inconsistent with the principles of recovery‐oriented care (Weeghel et al. 2019). Psychiatric nurses must therefore balance recovery‐oriented care with the implementation of necessary coercive interventions, creating inherent role conflicts. Although Geller (2012) acknowledged that coercive interventions may be necessary to ensure service user safety and facilitate recovery, he emphasised that they should be implemented in a humane and respectful manner that aligns with recovery goals.

Previous studies have demonstrated that the quality of nurse–service user interactions substantially influences service users' experiences of coercion. When coercive interventions are accompanied by humane treatment, respectful interactions, and clear communication, service users perceive these measures less negatively (Aguilera‐Serrano et al. 2018). A recent systematic review further highlighted that open communication and therapeutic engagement can mitigate the adverse effects of coercive interventions. Effective strategies include explaining the reasons for and expected duration of the intervention and providing opportunities for service users to participate in decision‐making. These approaches primarily reduce perceptions of coercive interventions as nontherapeutic, dehumanising, or an unjustified exercise of power (Griffin et al. 2025).

Nevertheless, negative attitudes or emotions toward service users, including fear and stigma, may be reflected in discriminatory or avoidant interpersonal care, thereby reinforcing paternalistic or dominance‐oriented practices (Paterson et al. 2013). Attitudes, defined as relatively stable evaluations ranging from negative to positive (Dickens et al. 2022), play a critical role in guiding nursing practice, particularly regarding coercive interventions. Although attitudes represent internal evaluations, the behavioural patterns nurses exhibit during interactions, such as dominance and coldness, are referred to as interpersonal care characteristics. This concept provides a framework for understanding how nurses interact with service users in clinical practice (Kinoshita and Shimosato 2020). Interpersonal care characteristics are conceptualised using the interpersonal circumplex (IPC) model, which classifies interpersonal behaviour along two dimensions: dominance (superiority–inferiority) and coldness (cold–warm characteristics). Dominance reflects assertiveness, management, or control, whereas coldness reflects lower levels of empathy and emotional support (Kinoshita and Shimosato 2020). Although attitudes toward coercive interventions are multidimensional and complex (Dickens et al. 2022), the IPC model provides a useful framework for examining how these attitudes may be expressed in caregiving behaviours.

Research explicitly examining the associations between nurses' attitudes and interpersonal care characteristics remains limited (Dickens et al. 2022). Lambert et al. (2018) quantitatively investigated the relationship between nurses' attitudes toward coercion and their boundary management styles (controller, pacifier, and negotiator). They reported positive correlations between favourable attitudes toward coercion and both the pacifier and controller styles. However, these attitudes did not significantly predict behaviour in multiple regression analyses. Although theoretical frameworks suggest that recovery‐oriented care promotes more positive perceptions of service users and reduces the use of coercive interventions (Lim et al. 2019; Sreeram et al. 2021), empirical evidence clarifying these associations remains limited.

Addressing this gap in the literature has important implications for psychiatric nursing practice, particularly regarding how internal attitudes translate into specific caregiving behaviours during coercion. Without endorsing the use of coercive interventions, examining the cross‐sectional associations among recovery‐oriented attitudes, perceptions of coercive interventions, and interpersonal care characteristics provides an evidence base for concrete practical applications. Specifically, identifying the mechanisms by which recovery beliefs reduce stigma‐based evaluations and subsequent interpersonal coldness can directly inform routine psychiatric nursing education. Furthermore, understanding these associations may complement established approaches, including trauma‐informed care and psychiatric advance directives (Substance Abuse and Mental Health Services Administration 2014; Swanson et al. 2000).

## 2 Background

This study proposes a conceptual model in which nurses' recovery‐oriented attitudes are associated with their attitudes toward coercive interventions (Hypothesis 1), which, in turn, are associated with interpersonal care characteristics, particularly the dimensions of dominance and coldness (Hypothesis 2). Figure 1 illustrates the hypothesised relationships.

Hypothesis 1 *Nurses with stronger recovery‐oriented attitudes are more likely to perceive coercive interventions as undesirable*.

**FIGURE 1:**
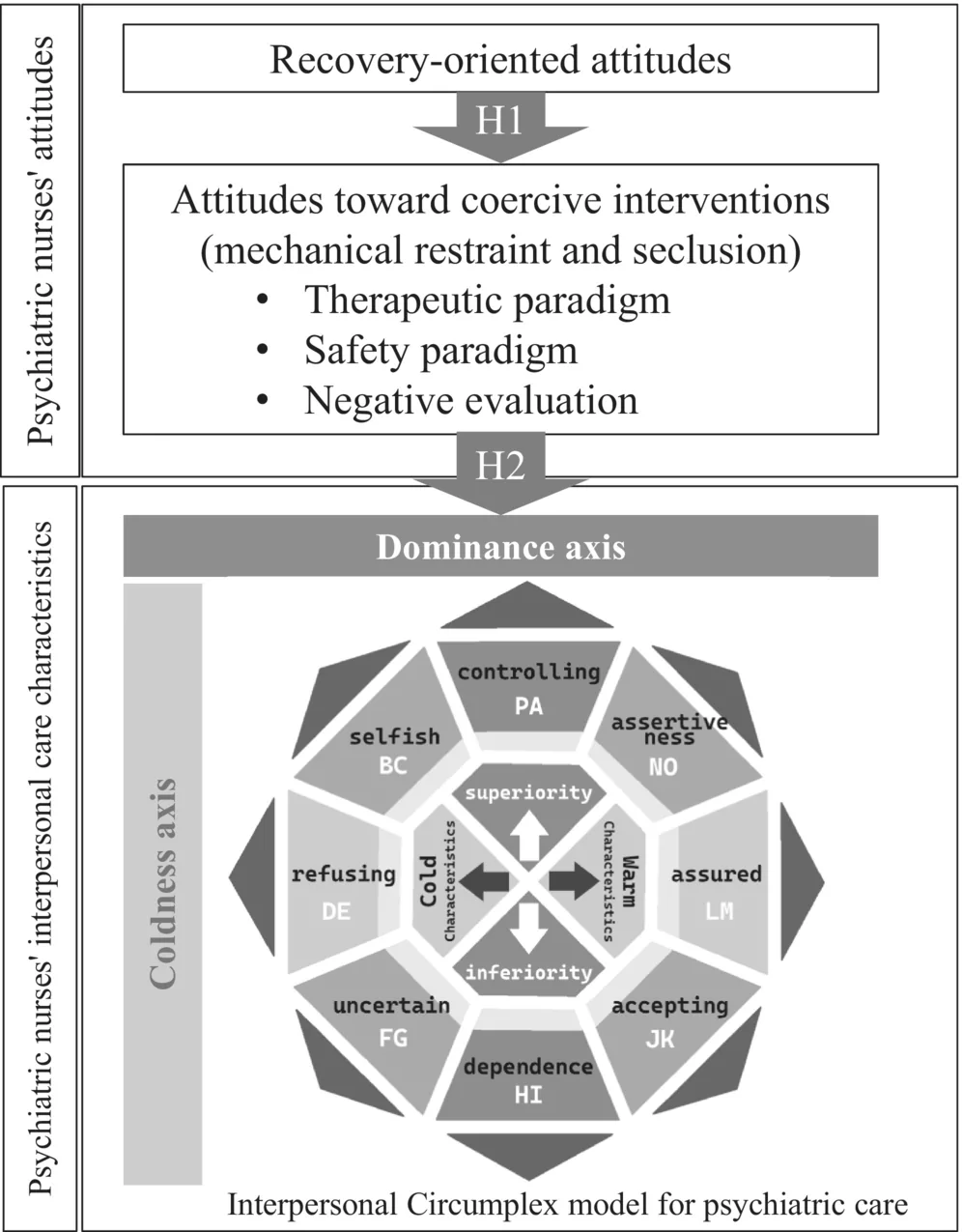
Conceptual framework. Hypothesis 1: Nurses with stronger recovery‐oriented attitudes are more likely to perceive coercive interventions as undesirable. Hypothesis 2: Negative attitudes toward coercive interventions are expected to be associated with lower levels of dominance and coldness in interpersonal care. Therapeutic paradigm: The belief that coercive interventions (e.g., restraint and seclusion) facilitate recovery and provide therapeutic benefits for service users (Kinoshita and Shimosato 2022; Doedens et al. 2020). Safety paradigm: The belief that coercive interventions, although potentially harmful, are sometimes necessary to ensure the safety of service users and staff (Kinoshita and Shimosato 2022; Doedens et al. 2020). Negative evaluation: Negative judgmental attitudes toward service users, such as perceiving them as aggressive, unpredictable, or incapable of understanding staff communication (Kinoshita and Shimosato 2022). Interpersonal Circumplex Model for Psychiatric Care (IPC‐PC) (Kinoshita and Shimosato 2020): This model is based on two dimensions: the vertical axis represents dominance, ranging from superiority to inferiority, whereas the horizontal axis represents coldness, ranging from cold to warm characteristics. Together, these dimensions define eight interpersonal factors spanning PA to NO. For example, the BC (selfish) characteristic combines the superiority dimension represented by PA and the coldness dimension represented by DE, demonstrating positive correlations with both. To minimise multicollinearity, this study focused on four primary interpersonal characteristics derived from these two dimensions: dominance (superiority–inferiority) and coldness (cold–warm characteristics).

Although coercive interventions are frequently justified as essential for maintaining safety (Doedens et al. 2020), maintaining recovery‐oriented attitudes under these circumstances remains challenging but achievable (Lorien et al. 2020). Recovery‐oriented attitudes encompass two complementary dimensions: an optimistic belief that recovery is possible and a realistic recognition of the challenges involved (Borkin et al. 2000; Chiba et al. 2016). Nurses who embrace recovery‐oriented principles, including autonomy, equality, and strengths‐based care, may be more sensitive to the ethical tensions associated with coercive interventions. Consequently, they may be less likely to endorse coercive interventions or hold negative evaluations of service users.

Hypothesis 2 *Negative attitudes toward coercive interventions are expected to be associated with lower dominance and coldness in interpersonal care*.

Previous research has highlighted the influence of nurses' attitudes and personal characteristics on the use of coercive interventions (Dickens et al. 2022; Lambert et al. 2018). Viewing coercive interventions through therapeutic or safety paradigms (Doedens et al. 2020) may reinforce paternalistic and dominance‐oriented approaches to care (Paterson et al. 2013). In contrast, nurses who question or reject coercive interventions may be likely to engage collaboratively with service users through shared decision‐making and clear communication (Griffin et al. 2025). Based on this evidence, we hypothesised that more negative attitudes toward coercive interventions would be reflected in interpersonal care characterised by lower levels of dominance and coldness.

## 3 The Study

### 3.1 Aims and Objectives

This study aimed to examine the associations among hospital‐based psychiatric nurses' recovery‐oriented attitudes, their attitudes toward coercive interventions, and their interpersonal care characteristics, particularly the dimensions of dominance and coldness. By empirically testing these hypothesised associations, this study sought to provide foundational evidence to support recovery‐oriented psychiatric nursing practice in the context of coercive interventions.

## 4 Methodology

### 4.1 Design

This quantitative, hypothesis‐testing study adhered to the Strengthening the Reporting of Observational Studies in Epidemiology guideline for cross‐sectional studies.

### 4.2 Instruments: Validity and Reliability

#### 4.2.1 Basic Attributes

The following participant characteristics were collected: sex, age, years of nursing experience, years of psychiatric nursing experience, department, and awareness of recovery. Age was categorised by decade to reduce respondents' potential reluctance to report their exact age. Awareness of recovery was assessed using a 4‐point scale, with a score of 1 indicating ‘not knowledgeable’ and a score of 4 indicating ‘very knowledgeable.’

#### 4.2.2 Recovery‐Oriented Attitudes

Nurses' recovery‐oriented attitudes were assessed using the Japanese version of the Recovery Attitude Questionnaire (RAQ‐7) (Chiba et al. 2016), which was adapted from the original RAQ‐7 developed by Borkin et al. (2000). The RAQ‐7 comprises seven items across two factors: four items assess ‘Recovery is possible and needs faith’, whereas three items assess ‘Recovery is difficult and differs among people’. Responses are rated on a 5‐point scale ranging from 1 (not at all agree) to 5 (strongly agree), with higher scores indicating more positive recovery‐oriented attitudes.

The Japanese version demonstrated acceptable construct validity (goodness‐of‐fit index (GFI), adjusted goodness‐of‐fit index (AGFI), Comparative Fit Index (CFI) > 0.90, root mean square error of approximation (RMSEA) < 0.05) and moderate internal consistency (Cronbach's *α* = 0.53 and 0.56), with a mean inter‐item correlation of 0.64 (Chiba et al. 2016).

#### 4.2.3 Nurses' Attitudes Toward Coercive Intervention

Nurses' attitudes toward coercive interventions were assessed using the Japanese version of the Ethical Perceptions of Physical Restraint Scale (EPPRS) (Kinoshita and Shimosato 2022). In Japan, however, training in manual restraint is not mandatory, and some psychiatric nurses may therefore have limited knowledge and experience with this practice. Because seclusion and mechanical restraint represent the primary coercive interventions used in Japanese psychiatric settings (Savage et al. 2024), the EPPRS was adapted for this study by replacing references to physical restraint with seclusion and mechanical restraint. This modified version has not yet undergone psychometric validation and should therefore be interpreted with caution. Furthermore, in Japan, only psychiatrists are authorised to initiate or discontinue these interventions. Although the internationally recognised Staff Attitude to Coercion Scale is available, no validated Japanese version existed at the time of this study; therefore, the EPPRS was selected.

The EPPRS comprises three factors: the ‘therapeutic paradigm’ (three items), reflecting beliefs that seclusion and mechanical restraint have therapeutic effects; ‘safety paradigm’ (six items), reflecting acceptance of coercive interventions as necessary to maintain safety; and ‘negative evaluations’ (five items), reflecting negative perceptions of service users as difficult or incapable of adequate self‐regulation. The modified 17‐item scale (three items excluded from the original version) is rated on a 7‐point Likert scale ranging from 1 (‘not at all’) to 7 (‘strongly agree’), with higher scores indicating greater acceptance of or resignation toward coercive interventions. Reported internal consistency (Cronbach's *α*) for the three subscales ranged from 0.51 to 0.76 (Kinoshita and Shimosato 2022). The scale was originally developed in Japanese, and the English names of the factors are presented with permission from the original author.

#### 4.2.4 Interpersonal Care Characteristics

Psychiatric nurses' interpersonal care characteristics were assessed using the Japanese version of the IPC‐PC (Kinoshita and Shimosato 2020). The IPC‐PC is an eight‐sector IPC organised around two primary dimensions: dominance (superiority–inferiority) and coldness (cold–warm characteristics). The eight interpersonal factors are designated by the letter pairs PA, BC, DE, FG, HI, JK, LM and NO, which were created by combining consecutive letters from A to P in a counterclockwise sequence (Leary 1957; Kinoshita and Shimosato 2020). These labels are identifiers rather than abbreviations and should not be expanded.

Within the IPC‐PC, PA (controlling) and HI (dependence) represent the core characteristics of superiority and inferiority, respectively, along the dominance dimension. Similarly, DE (refusing) and LM (assured) represent cold and warm characteristics, respectively, along the coldness dimension. The remaining four factors—BC (selfish), FG (uncertain), JK (accepting), and NO (assertiveness)—represent intermediate interpersonal characteristics that combine elements of both dominance and coldness.

The IPC‐PC is a 32‐item, eight‐factor self‐report instrument scored on a 5‐point Likert scale ranging from 1 (‘not at all true’) to 5 (‘very true’). Reported Cronbach's *α* coefficients for the eight subscales range from 0.51 to 0.79 (Kinoshita and Shimosato 2020), indicating acceptable internal consistency.

### 4.3 Sampling and Recruitment

A cross‐sectional survey was conducted between August and October 2022 among nurses employed at nine psychiatric hospitals with forensic psychiatric units in the Kanto region, Japan. In Japan, forensic psychiatric units are established only in public hospitals that satisfy the criteria established by the Ministry of Health, Labour and Welfare. After obtaining permission from the nursing directors by telephone, survey packets containing the questionnaires and return envelopes were mailed to each hospital, and the nursing directors coordinated their distribution.

### 4.4 Sample Size and Power

A post hoc power analysis was conducted for the proposed structural equation model (SEM) using the RMSEA criterion and the web‐based utility (http://quantpsy.org/) developed by Preacher and Coffman (2006). Based on a sample size of 604, degrees of freedom (df) = 61, null RMSEA_0_ = 0.05, and alternative RMSEA_a_ = 0.065, the estimated statistical power was 0.82, indicating adequate power to detect the hypothesised model.

### 4.5 Quality Appraisal

Although this study was limited to public hospitals in the Kanto region, all hospitals with forensic psychiatric units within the region were included, providing complete coverage of the target population in that setting. This sampling strategy minimised regional sampling bias; however, the findings may not be generalisable to other regions. The response period was limited to 2 weeks, which is considered appropriate for paper‐based surveys conducted in clinical settings. No financial incentives were provided, and no formal reminder notices were issued because of institutional constraints. Nevertheless, the final sample size was sufficient for the planned analyses, and respondents' demographic characteristics, including age, sex, and years of psychiatric nursing experience, were broadly comparable with those reported in previous national surveys (e.g., Kinoshita and Shimosato 2020; Mukaihara et al. 2019), supporting the representativeness of the sample.

### 4.6 Data Abstraction

Survey responses were anonymised and converted into analysable numerical data.

Based on Leary's (1957, 69) scoring method for dominance and warmth in the IPC model [Dominance = PA − HI + 0.7(NO + BC − FG − JK); Warmth = LM − DE + 0.7(NO − BC − FG + JK)], we developed an original scoring method to derive four indices: superiority, inferiority, cold characteristics, and warm characteristics. This modification was introduced because Leary's (1957) formula may not adequately represent situations in which superiority and inferiority are simultaneously elevated.

Superiority = PA + 0.7(BC + NO).

Inferiority = HI + 0.7(FG + JK).

Cold characteristics = DE + 0.7(BC + FG).

Warm characteristics = LM + 0.7(JK + NO).

Descriptive statistics were calculated for all variables. Response validity was evaluated by identifying univariate outliers (|*Z*| ≥ 3) and multivariate outliers using Mahalanobis distance evaluated at *p* < 0.001.

Although the Shapiro–Wilk test indicated significant departures from normality, skewness (≤ ±0.70) and kurtosis (≤ ±0.92) remained within acceptable limits (Curran et al. 1996). Considering these values and our adequate sample size, the use of parametric statistical analyses was considered appropriate (Kim 2013).

The internal consistency (Cronbach's α) of the RAQ‐7, EPPRS, and IPC‐PC ranged from 0.55 to 0.77, consistent with values reported in previous studies (Table 1).

**TABLE 1:** Basic characteristics of the included nurses.

	*n*	%			Max–Min	Mean	SD	Cronbach's *α*
**Gender (*n* = 602)**			**Years of experience**					
Male	227	37.7		Years of nursing experience (*n* = 598)	1–43	18.98	10.51	
Female	374	62.1		Years of psychiatric nursing experience (*n* = 601)	0–43	13.51	9.04	
Refused to answer	1	0.20						
**Age (*n* = 603)**			**IPC‐PC‐J (the Interpersonal Circumplex model for Psychiatric Care)**					
20–29	72	11.9		PA ‘controlling’	4–20	12.38	2.54	0.74
30–39	108	17.9		BC ‘selfish’	4–17	9.88	2.19	0.70
40–49	226	37.5		DE ‘refusing’	4–19	10.40	2.57	0.77
50–59	172	28.5		FG ‘uncertain’	4–20	10.82	2.64	0.77
≥ 60	25	4.1		HI ‘dependence’	4–20	11.20	2.76	0.72
**Job title (*n* = 595)**				JK ‘accepting’	6–20	13.52	2.31	0.76
Head nurse or higher	33	5.5		LM ‘assured’	6–20	13.97	2.26	0.74
Deputy head nurse	198	32.8		NO ‘assertiveness’	7–20	14.29	2.01	0.55
Staff nurse	364	60.3	**EPPRS‐J (the Ethical Perceptions of Physical Restraint Scale)**					
**Department (*n* = 579)**				Therapeutic paradigm	3–21	13.48	3.34	0.68
Forensic psychiatric wards	174	28.8		Safety paradigm	7–42	27.70	5.72	0.75
Acute psychiatric wards	105	17.4		Negative evaluations	5–33	15.80	4.94	0.74
Other psychiatric wards	175	29.0						
Community support	40	6.6						
Other wards (e.g., physical wards)	85	14.1	**RAQ‐7 (the Recovery Attitude Questionnaire)**					
**Knowledge about recovery (*n* = 600)**				Recovery is possible and needs faith	10–20	14.87	1.85	0.58
Very knowledgeable	28	4.6		Recovery is difficult and differs among people	6–15	11.98	1.43	0.69
Mostly knowledgeable	265	43.9						
Slightly knowledgeable	266	44.3						
Not knowledgeable	41	6.8						

*Note:* Although the EPPRS was originally developed to assess attitudes toward manual restraint, the items in this study were adapted to refer specifically to seclusion and mechanical restraint. This adapted version has not yet undergone formal validation.

Little's Missing Completely at Random (MCAR) test was significant (*χ* ^2^ = 2603.82, *p* = 0.008), indicating rejection of the MCAR assumption. Because MCAR could not be assumed and missing at random (MAR) cannot be directly tested using observed data, multiple imputation (100 imputed datasets) was performed under a plausible MAR assumption (Sterne et al. 2009). Considering the low overall proportion of missing data (≤ 5%), sensitivity analyses using listwise deletion and mean/mode imputation were also conducted to evaluate the robustness of the findings across alternative approaches to handling missing data. All approaches produced highly consistent results with minimal variation. Specifically, *t*‐values differed by ≤±0.50 (*d* ≤ ±0.03), analysis of covariance (ANCOVA) *B* values differed by ≤±0.38 (partial *η* ^2^ ≤ ±0.003). These results indicate that the substantive findings were robust across alternative approaches to handling missing data.

### 4.7 Population and Sample

Of the 1715 questionnaires distributed, 613 were returned, yielding a response rate of 35.7%. After excluding responses identified as outliers based on *Z*‐scores, Mahalanobis distance, and implausible response patterns (e.g., selecting only the maximum or minimum response for all items), 604 valid questionnaires were included in the analysis. Specifically, six cases were excluded because of missing data, one case because of identical responses, and two cases as extreme multivariate outliers, corresponding to a valid response rate of 98.50% (Figure 2).

**FIGURE 2:**
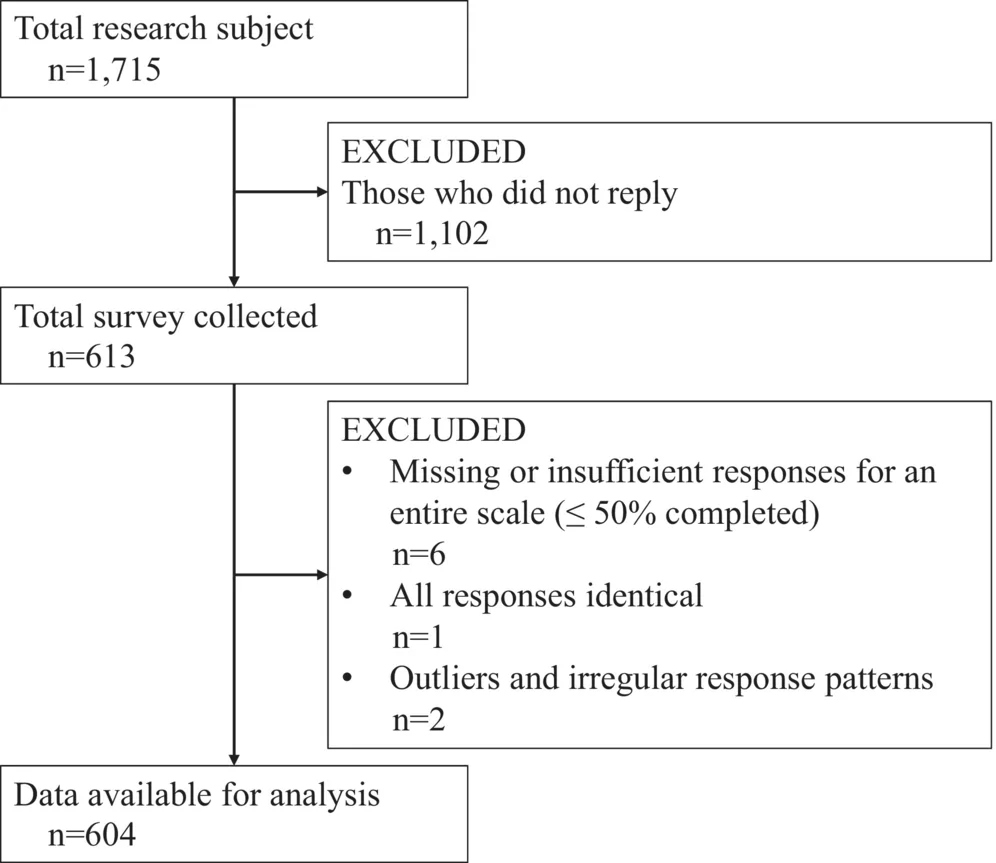
Overview of the study setting.

### 4.8 Inclusion and/or Exclusion Criteria

The inclusion criteria comprised nurses employed at public psychiatric hospitals with forensic psychiatric units in the Kanto region, Japan. The exclusion criteria included questionnaires containing outlier responses, implausible response patterns, or incomplete data.

### 4.9 Data Collection

Participants received written information describing the study, including its purpose and procedures, and were asked to complete and return the questionnaires within 2 weeks.

### 4.10 Data Analysis

Independent‐samples *t*‐tests were performed to compare groups by sex and recovery knowledge (knowledgeable vs. not knowledgeable). ANCOVA was conducted to examine differences according to job title and department. Departments were categorized as forensic psychiatric wards, acute psychiatric wards, and community support services. Other psychiatric ward types were excluded because of their heterogeneity. To control for Type I error associated with multiple comparisons, a Bonferroni correction was applied to the *t*‐tests and ANCOVAs. Considering the 18 independent tests conducted within each analysis family, the statistical significance threshold was adjusted to *p* < 0.0028.

SEM was performed to examine the associations among recovery‐oriented attitudes, attitudes toward coercive interventions, and interpersonal care characteristics. Variables were initially selected based on preliminary Pearson's correlation analyses and the study's theoretical and conceptual framework, with items retained according to their conceptual relevance to the hypothesised model.

Model fit was evaluated using the CFI (> 0.90), Tucker–Lewis Index (TLI) (> 0.90), and RMSEA (< 0.05). The model demonstrating the best overall fit was selected. All statistical analyses were performed using IBM SPSS Statistics version 27.0 and IBM SPSS Amos version 29.0.

### 4.11 Ethical Considerations

This study was approved by the Committee for Medical Ethics of Shinshu University. Participants received written information describing the study's purpose, voluntary nature, anonymity, data management procedures, and publication plans. Return of the completed questionnaire was considered to indicate implied informed consent. To protect confidentiality, completed questionnaires were returned in sealed envelopes. Data were collected anonymously, and no responses could be linked to individual participants. Paper records were stored securely, and electronic data were protected through restricted access.

## 5 Results

### 5.1 Subjects

Most participants were women (62.1%) and were in their 40s, with a mean of 13.51 ± 9.04 years of psychiatric nursing experience. The most common job title was staff nurse, followed by deputy head nurse and head nurse or higher. Participants were most frequently assigned to forensic psychiatric wards and psychiatric emergency wards. On average, years of overall nursing experience slightly exceeded years of psychiatric nursing experience. The sample was nearly evenly divided between nurses who reported being ‘knowledgeable’ about recovery (*n* = 293) and those who reported ‘not knowledgeable’ (*n* = 307) (Table 1).

### 5.2 Preliminary Analyses of Group Differences and Correlates

Independent‐samples *t*‐tests revealed no significant differences between men and women after Bonferroni correction. Comparisons between the ‘knowledgeable’ and ‘not knowledgeable’ recovery groups revealed significant differences across multiple variables. The knowledgeable group scored lower on the ‘safety paradigm’ (*t* = 3.08, *p* = 0.002, *d* = 0.253) and ‘negative evaluations’ (*t* = 3.60, *p* < 0.001, *d* = 0.294). They also scored higher on the recovery‐oriented attitude subscales, ‘Recovery is possible and needs faith’ (*t* = −5.65, *p* < 0.001, *d* = −0.463) and ‘Recovery is difficult and differs among people’ (*t* = −4.40, *p* < 0.001, *d* = −0.359). Furthermore, the knowledgeable group reported lower ‘cold’ characteristics (*t* = 7.07, *p* < 0.001, *d* = 0.579) and higher ‘warm’ characteristics (*t* = −8.14, *p* < 0.001, *d* = 0.668).

ANCOVA results using job title as the predictor indicated that staff nurses scored significantly higher on ‘negative evaluations’ than deputy head nurses (*B* = −1.70, *p* < 0.001, partial *η* ^2^ = 0.026). For interpersonal care characteristics, staff nurses also showed higher ‘cold characteristics’ than head nurses (*B* = −2.80, *p* < 0.001, partial *η* ^2^ = 0.022). ANCOVA results using department (forensic psychiatric wards, acute psychiatric wards, and community support services) as the independent variable demonstrated that nurses in forensic psychiatric wards scored significantly higher on the ‘safety paradigm’ (B = 3.43, *p* = 0.001, partial *η* ^2^ = 0.031) and ‘negative evaluations’ (*B* = 3.20, *p* = 0.001, partial *η* ^2^ = 0.042) than those in community support services.

Overall, the observed effect sizes ranged from small to moderate, indicating meaningful differences in recovery‐oriented attitudes, attitudes toward coercive interventions, and interpersonal care characteristics.

### 5.3 Associations Among Recovery‐Oriented Attitudes, Attitudes Toward Coercive Interventions, and Interpersonal Care Characteristics

Based on the hypothesised conceptual model (Figure 1), a SEM was developed to examine the associations among psychiatric nurses' recovery‐oriented attitudes, attitudes toward coercive interventions, and interpersonal care characteristics (Figure 3). The model demonstrated an acceptable overall fit (*χ* ^2^ = 215.719, df = 61, *p* < 0.001; TLI = 0.91, CFI = 0.95, and RMSEA = 0.065). The latent variables (‘Recovery‐oriented attitudes,’ ‘Resignation,’ and ‘Stigma‐based evaluation’) were refined to improve conceptual clarity and theoretical coherence.

**FIGURE 3:**
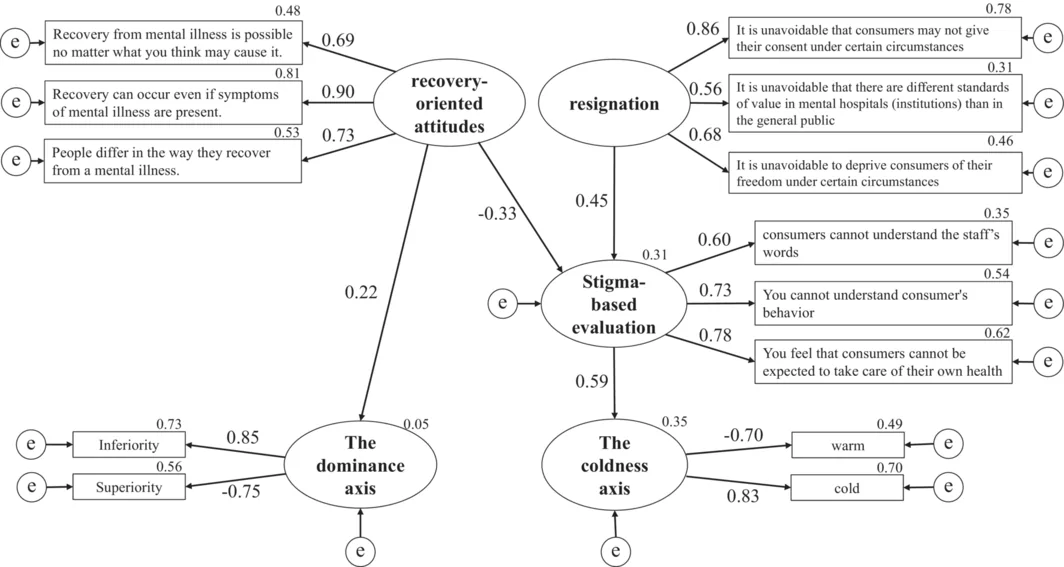
Results of the structural equation modelling. Recovery‐oriented attitudes were represented by two items from the Recovery Attitudes Questionnaire (RAQ‐7) subscale ‘Recovery is possible and needs faith’ and one item from the subscale ‘Recovery is difficult and differs among people’. Together, these items represent the belief that recovery is possible while recognising that the recovery process varies among individuals. Resignation was represented by three items from the ‘safety paradigm’ subscale of the Ethical Perceptions of Physical Restraint Scale (EPPRS), reflecting acceptance of the perceived necessity of coercive interventions, including seclusion and mechanical restraint. Stigma‐based evaluation was represented by three items from the ‘negative evaluation’ subscale of the EPPRS, reflecting a tendency to focus on service users' perceived deficits and to make critical judgements about them.

Regarding Hypothesis 1, the relationships between recovery‐oriented attitudes and attitudes toward coercive interventions were examined. The findings partially supported the hypothesis: Higher recovery‐oriented attitudes and lower ‘Resignation’ (acceptance of coercive interventions as unavoidable) were both significantly associated with lower stigma‐based evaluations of service users.

Regarding Hypothesis 2, the associations between these variables and interpersonal care characteristics were examined across the two primary interpersonal dimensions.

On the dominance dimension, recovery‐oriented attitudes were positively associated with the latent dominance construct (standardised path coefficient = 0.22). Because superiority had a negative factor loading and inferiority had a positive factor loading, this relationship reflects a tendency toward a less dominant interpersonal style, characterised by lower superiority and higher inferiority. Although the model explained only 5% of the variance in the dominance construct, this latent variable was retained to preserve the theoretical integrity of the hypothesised model. Excluding the dominance construct improved overall model fit (χ^2^ = 92.105, df = 41, *p* < 0.001, TLI = 0.96, CFI = 0.98, RMSEA = 0.045); however, it was retained because of its conceptual importance to the proposed theoretical framework.

On the coldness dimension, higher stigma‐based evaluation of service users was associated with higher coldness scores, reflecting greater cold characteristics and fewer warm characteristics during interpersonal care. These findings were generally consistent with Hypothesis 2, indicating that more stigmatizing evaluations of service users were associated with less empathetic interpersonal care.

## 6 Discussion

### 6.1 Strengths and Limitations of the Work

#### 6.1.1 Relationship Between Recovery‐Oriented Attitudes and Attitudes Toward Coercive Interventions

The findings of this study partially support Hypothesis 1, which proposed that stronger recovery‐oriented attitudes would be associated with lower acceptance of coercive interventions. Specifically, recovery‐oriented attitudes were negatively associated with stigma‐based evaluations of service users, including perceptions that they are unpredictable and incapable of communicating effectively or managing their own health. Such perceptions may reinforce harmful stereotypes, undermine mutual understanding and self‐management, and contribute to pessimistic expectations regarding recovery, particularly among staff caring for individuals with severe mental illness (Ross and Goldner 2009). These perceptions may also serve to justify the use of coercive interventions (Paterson et al. 2013). Thus, the present findings are broadly consistent with previous research linking recovery‐oriented perspectives to less favourable attitudes toward coercion, while suggesting that perceptions of service users may be an important factor in this relationship.

Lower levels of resignation were independently associated with lower stigma‐based evaluations. However, no direct association was observed between recovery‐oriented attitudes and resignation, suggesting that resignation may reflect learned helplessness arising from repeated exposure to challenging clinical situations and contextual factors beyond the individual level (Peterson et al. 1993). Because decisions regarding the initiation, authorisation, and implementation of coercive interventions are strongly influenced by organisational culture (Pawlowski and Baranowski 2018), resignation may be shaped more by organisational culture and repeated exposure to challenging clinical situations than by individual recovery‐oriented attitudes. Thus, reducing resignation may require organisational and cultural changes alongside individual‐level education.

Importantly, nurses who reported greater knowledge of recovery were less likely to endorse coercive interventions. This finding is consistent with Anthony's (1993) conceptualisation of recovery as a nonlinear, person‐centred process supported by empathy and noncoercive engagement, as well as with evidence demonstrating that staff attitudes influence perceptions of coercion (Aguilera‐Serrano et al. 2018; Griffin et al. 2025). Alongside the association between recovery‐oriented attitudes and stigma‐based evaluations, these findings extend previous research by integrating recovery knowledge, perceptions of service users, and attitudes toward coercive interventions within a single structural model. This integrated perspective is particularly relevant in Japan, where recovery‐oriented psychiatric care continues to develop (Matsui et al. 2023).

#### 6.1.2 Relationship Between Recovery Attitudes and Dominance

The study hypothesised that recovery‐oriented attitudes would be associated with the dominance dimension of interpersonal care. Within the IPC‐PC, dominance (superiority–inferiority characteristics) represents the power dimension of interpersonal relationships and reflects an individual's exercise of power to influence others (Birtchnell 1993). However, only 5% of the variance in the dominance axis was explained, suggesting that it contributed relatively little to the overall model.

This limited explanatory power may partly reflect characteristics of the measurement model. Specifically, the NO (assertiveness) subscale, which contributes to both the dominance (Inferiority) and coldness (warm characteristics) dimensions, demonstrated lower internal consistency (*α* = 0.55) than the remaining subscales (*α* = 0.70–0.77). Measurement error in this shared component may therefore have attenuated the observed path coefficients involving these latent constructs.

Beyond measurement considerations, the conceptual nature of dominance may also explain these findings. Superiority may represent a directive or controlling interpersonal style supported by professional authority, whereas inferiority may reflect insecurity or fear that increases reliance on coercive interventions for self‐protection. These opposing tendencies may partially offset one another statistically, thereby reducing the explanatory power of the overall dominance construct.

The limited contribution of superiority contrasts with previous research emphasising professional superiority and power imbalances as barriers to recovery‐oriented practice (Slade et al. 2014; Molina‐Mula and Gallo‐Estrada 2020). Slade et al. (2014) argued that excessive professional dominance may hinder recovery‐oriented practice; Molina‐Mula and Gallo‐Estrada (2020) suggested that nurses often prefer submissive and passive service users. Such preferences may reinforce power imbalances, diminish service users' autonomy, and encourage judgements based primarily on compliance. This discrepancy may reflect differences in how these constructs are conceptualised and measured. Although previous research has emphasised professional authority and power imbalance, the IPC‐PC dominance dimension incorporates both superiority and inferiority characteristics. Birtchnell (1993) conceptualised inferiority as attitudes and behaviours involving the pursuit or receipt of approval, support, or direction from others, rather than simply passivity. In clinical practice, these characteristics may involve seeking service users' approval or acceptance to encourage cooperation with care, which may appear submissive while also reflecting attempts to influence behaviour. Thus, recognising these complex power dynamics may be relevant to recovery‐oriented care, which emphasises service users' autonomy and participation.

#### 6.1.3 Relationship Between Recovery Attitudes and Coldness

In the SEM, the items representing ‘therapeutic paradigm,’ which conceptualises seclusion and mechanical restraint as therapeutic interventions, were excluded. This finding may reflect the increasing prominence of the ‘safety paradigm’ (Doedens et al. 2020) and nurses' generally unfavourable attitudes toward coercive interventions (Bregar et al. 2018). The construct resignation identified in this study represents the belief that coercive interventions are inevitable, potentially reflecting persistent value conflicts between broader societal expectations and psychiatric practice. In Japan, structural and cultural norms may reinforce this perspective by framing coercive interventions as an essential component of psychiatric care and encouraging passive acceptance. Comparable patterns have been reported internationally, particularly in settings where coercive interventions remain common because effective noncoercive alternatives have not been fully established or integrated into routine practice (Galbert et al. 2023; Laukkanen et al. 2019).

Importantly, stronger recovery‐oriented attitudes were associated with lower levels of stigma‐based evaluations of service users, which, in turn, were associated with lower interpersonal coldness. This latter association is consistent with previous research linking negative perceptions of service users with greater interpersonal coldness (Ross and Goldner 2009). The present study extends this literature by showing that recovery‐oriented attitudes were associated with both these perceptions and interpersonal responses.

This finding is consistent with Geller's (2012) assertion that, even when coercive interventions are unavoidable, they should be implemented in a humane and respectful manner. Thus, promoting recovery‐oriented attitudes through education and opportunities to observe service users' recovery may help reduce stigmatizing evaluations and foster more respectful interpersonal care. These findings underscore the importance of education and organisational support in fostering compassionate, recovery‐oriented psychiatric nursing (Chiba et al. 2019).

#### 6.1.4 Limitations

This study has several limitations. First, participants were recruited exclusively from public hospitals to ensure the inclusion of nurses working in forensic psychiatric wards. However, because the participants' demographic characteristics were comparable to those reported in studies conducted in private hospitals, this sampling strategy is unlikely to have substantially influenced the findings.

Second, the response rate was 35.7%, which is typical for mail‐based surveys. Nevertheless, the possibility of nonresponse bias cannot be excluded. Nurses with greater interest in recovery‐oriented care or more favourable recovery‐oriented attitudes may have been more likely to participate. Consequently, the findings may slightly overestimate the prevalence of recovery‐oriented attitudes among psychiatric nurses.

Third, recovery knowledge was assessed using four broad self‐report categories (‘I know a lot about it’, ‘I know roughly about it’, ‘I don't know much about it’, and ‘I don't know’) to minimise respondent burden. However, this approach did not allow assessment of the accuracy or depth of participants' understanding, particularly considering the conceptual ambiguity surrounding the term ‘recovery’ (Slade et al. 2014). Future studies should employ more precise measures of recovery knowledge to better characterise its relationship with recovery‐oriented attitudes.

Fourth, methodological considerations concern the measurement instruments used in this study. Primarily, the modified version of the EPPRS used to assess attitudes toward seclusion and mechanical restraint has not undergone formal psychometric validation. Although this adaptation was necessary to reflect the specific clinical context in Japan, the lack of established structural validity warrants cautious interpretation of the findings. Furthermore, the internal consistency of several subscales (e.g., RAQ‐7 factors, IPC‐PC NO subscale, and some EPPRS factors) was modest (*α* = 0.51–0.55). Although these values are consistent with previous studies, this level of reliability may reduce the precision of the observed statistical relationships. In the present model, latent variables were refined within the SEM by retaining a subset of indicators to enhance conceptual clarity based on theoretical considerations. However, this item selection does not address the limited internal consistency of the original subscales, and the absence of cross‐validation with an independent sample means that model overfitting cannot be entirely excluded. Furthermore, the refined measurement model was not validated through a confirmatory factor analysis using an independent or split‐half sample. Consequently, although the refined resignation and stigma‐based evaluation constructs provided a conceptually meaningful representation within the present study, further validation using independent samples is needed to establish their factor structure, reliability, and generalisability.

Finally, this study was conducted exclusively in Japan, where recovery‐oriented practice remains less established and many psychiatric nurses report limited recovery knowledge. Although this context differs from countries in which recovery‐oriented care has been more extensively implemented, the observed associations between recovery‐oriented attitudes, stigma‐based evaluations, and interpersonal care characteristics may still have broader relevance. Nevertheless, replication in other cultural and health care settings is needed to determine the generalisability of these findings.

### 6.2 Implications for Nursing Practice

Integrating recovery‐oriented education into routine psychiatric nursing training may represent an important strategy for promoting empathetic, humane, and person‐centred care. The present findings are particularly relevant to the challenge of balancing recovery‐oriented practice with the realities of coercive psychiatric care. Service user‐led tools that support crisis self‐advocacy, such as psychiatric advance directives, are intended to strengthen autonomy but may be undermined by organisational cultures that favour coercive practices. As Swanson et al. (2000) noted, these autonomy‐promoting tools may paradoxically function as instruments of coercion when implemented within a paternalistic model of care.

The SEM provides preliminary insight into factors potentially associated with more recovery‐oriented interpersonal care. The findings suggest that specific recovery beliefs—particularly recognising recovery as a highly individualised process that remains possible regardless of diagnosis or ongoing symptoms—may help mitigate stigma‐based evaluations and subsequently lower interpersonal coldness. Because humane treatment, respect, and clear communication have been shown to reduce service users' negative experiences of coercive interventions (Aguilera‐Serrano et al. 2018), incorporating these recovery‐oriented principles into psychiatric nursing education may strengthen therapeutic relationships and help preserve service users' dignity and autonomy during situations in which coercive interventions cannot be avoided.

### 6.3 Recommendations for Further Research

Further research is needed to determine whether recovery‐oriented attitudes and lower stigma‐based evaluations are associated with clinical attitudes and practices that align with recovery‐oriented care in psychiatric inpatient settings. More robust measures of recovery knowledge are also needed to clarify its relationship with recovery‐oriented attitudes, stigma‐based evaluations, and attitudes toward coercive interventions.

Longitudinal and intervention studies are warranted to determine whether strengthening recovery‐oriented attitudes contributes to reductions in stigma and improvements in compassionate interpersonal care over time. Research conducted across a broader range of clinical settings, both within and outside Japan, is also necessary to evaluate the generalisability of the present findings. Furthermore, future studies should examine potential mediating and moderating factors, including organisational culture, leadership, and team dynamics, to better understand how recovery‐oriented principles are translated into routine psychiatric nursing practice.

## 7 Conclusions

Although recovery‐oriented attitudes were not directly associated with attitudes toward coercive interventions, stigma‐based evaluations emerged as a key construct within the proposed model; however, the association with the dominance dimension was comparatively limited. Differences in attitudes and interpersonal care characteristics were observed according to nursing experience, job title, and clinical department, underscoring the complexity of psychiatric nurses' perspectives.

More than half of the participants reported limited knowledge of recovery, suggesting that recovery‐oriented psychiatric care remains in an early stage of development in Japan. These findings suggest the potential value of strengthening recovery‐oriented attitudes through targeted education and organisational support to promote ethical, person‐centred psychiatric nursing while preserving service users' dignity when coercive interventions are unavoidable.

## Author Contributions

**Aimi Kinoshita:** writing – review and editing, conceptualization, methodology, software, validation, investigation, data curation, formal analysis, resources, project administration, visualization, writing – original draft, supervision. **Hisato Igarashi:** conceptualization, methodology, formal analysis, writing – original draft, writing – review and editing, data curation. **Shiori Ishida:** conceptualization, methodology, formal analysis, writing – original draft, writing – review and editing. **Seiji Shimosato:** conceptualization, data curation, formal analysis, writing – original draft, visualization, project administration, writing – review and editing, supervision, investigation, methodology, software, validation, funding acquisition, resources.

## Funding

This work was supported by the Japan Society for the Promotion of Science (JSPS) KAKENHI under the Grant‐in‐Aid for Scientific Research (C) (number 21K10680).

## Disclosure

Statistical Declaration: Statistical analyses for this study were conducted and verified by an expert statistician who is a co‐author of this manuscript (HI). The authors confirm that all statistical approaches were appropriately selected for the study design, implemented in accordance with the journal's statistical guidelines, and correctly interpreted.

## Ethics Statement

The study was conducted with the approval of the Committee for Medical Ethics of Shinshu University (Approval No. 5592, Date of Issue: July 29, 2022). The authors have checked to make sure that the submission conforms as applicable to the Journal's statistical guidelines described here. The authors affirm that the methods used in the data analyses are suitably applied to their data within the study design and context, and the statistical findings have been implemented and interpreted correctly. The authors agree to take responsibility for ensuring that the choice of statistical approach is appropriate and is conducted and interpreted correctly as a condition to submit to the journal.

## Conflicts of Interest

The authors declare no conflicts of interest.

## Data Availability

The data that support the findings of this study are available on request from the corresponding author. The data are not publicly available due to privacy or ethical restrictions.
